# Supplementary material for: New Insight into Short Time Exogenous Formaldehyde Application Mediated Changes in Chlorophytum comosum L. (Spider Plant) Cellular Metabolism
Source: Cells. 2023 Jan 5;12(2):232. doi: 10.3390/cells12020232 (PMC9857029; doi:10.3390/cells12020232)
Supplement: Supplementary file 1 [file cells-12-00232-s001.zip › cells-2064547-supplementary.pdf]

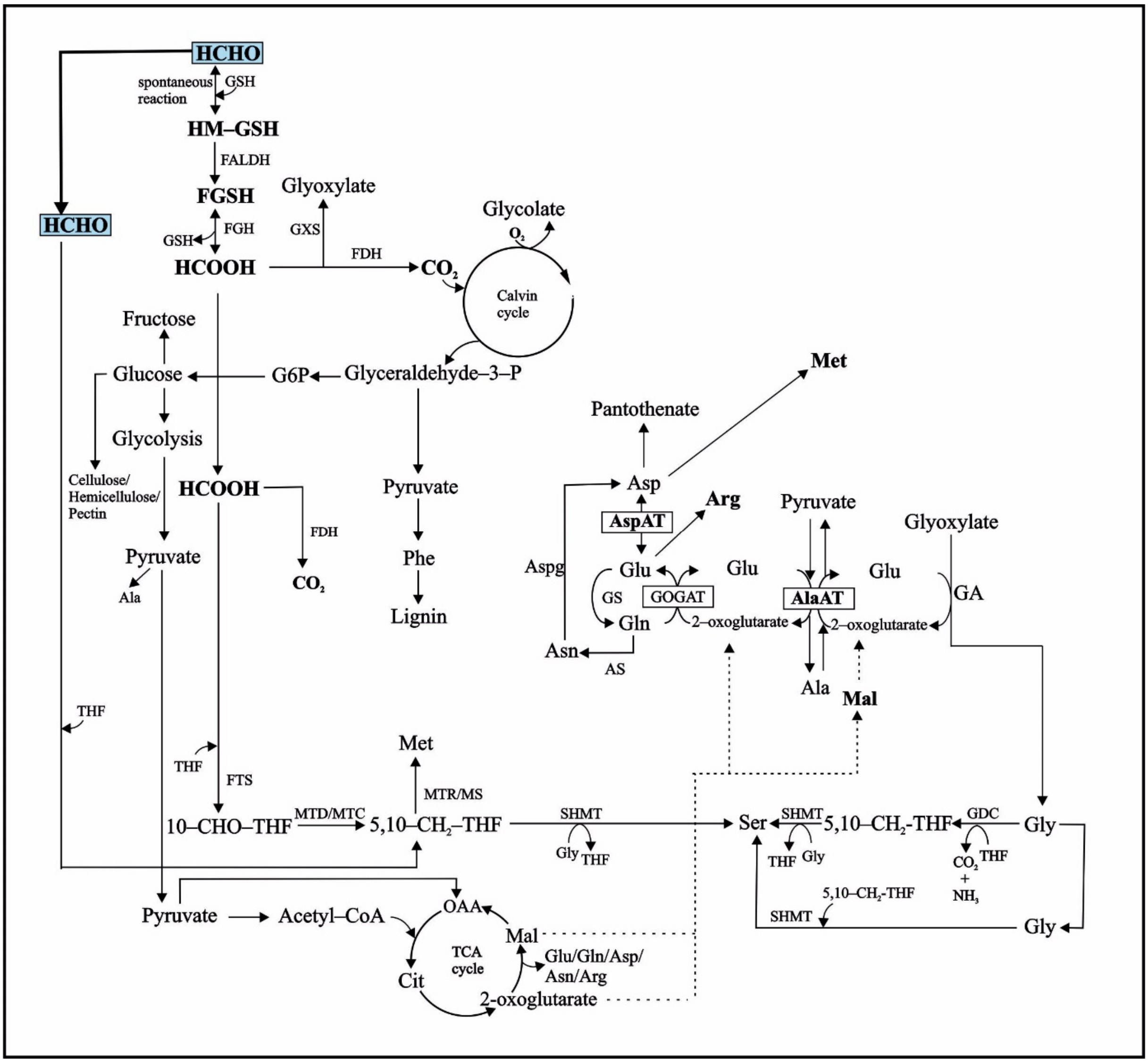

Figure S1. The possible processes of formaldehyde metabolism in plants. The abbreviations are as follows: HCHO: formaldehyde; HCOOH: formate; GSH: glutathione; HM-GSH: S-hydroxymethyl-glutathione; FALDH: GSH-dependent formaldehyde dehydrogenase; FGSH: S-formylglutathione; FGH: FGSH hydrolase; FDH: HCOOH dehydrogenase; THF: tetrahydrofolate; 10-CHO-THF: 10-formyl-THF; 5,10-CH_2-THF: 5,10-methylene-THF; FTS: 10-CHO-THF synthetase; MS: Met synthase; MTD/MTC: 5,10-CH_2-THF dehydrogenase/5,10-CH_2-THF cyclohydrolase; Met: methionine; Glu: glutamate; Gln: glutamine; Arg: arginine; Asp: aspartate; Asn: asparagine; Gly: glycine; GA: glyoxylate aminotransferase; Ser: serine; Cit: citrate; Ala: alanine; GXS: glyoxylate synthase; SHMT: serine hydroxymethyltransferase; GDC: glycine decarboxylase; OAA: oxaloacetate; Mal: malate; GS:glutamine synthetase; GOGAT: glutamine oxoglutarate aminotransferase; Aspg: asparaginase; AspAT: aspartate aminotransferase; AlaAT alanine aminotransferase; AS: asparagine synthase; GA: glyoxylate aminotransferase.

Table S1. Aeration time of the chamber to obtain a constant concentration of formaldehyde, i.e.20 mg $m^{-3.}$

**Aeration time**	**Concetration in air (mg m^{-3})**
Chamber without plants	
15 min.	10.975
30 min.	12.735
45 min.	15.709
60 min.	19.394
95 min.	20.101
110 min.	20.087
125 min.	20.171
12 h	20.2
24 h	20.33
48 h	20.23
Chamber with plants	
0 min.	20
48 h	0,02
